# *Quillaja* saponin mitigates methotrexate-provoked renal injury; insight into Nrf-2/Keap-1 pathway modulation with suppression of oxidative stress and inflammation

**DOI:** 10.1186/s40780-024-00330-4

**Published:** 2024-04-09

**Authors:** Mustafa Ahmed Abdel-Reheim, Merhan E. Ali, Ahmed Gaafar A. Gaafar, Ahmed Amine Ashour

**Affiliations:** 1 Department of Pharmaceutical Sciences, College of Pharmacy, Shaqra University, 11961 Shaqra, Saudi Arabia; 2 Department of Pharmacology and Toxicology, Faculty of Pharmacy, Beni-Suef University, Beni Suef, Egypt; 3 Department of Pathology, Faculty of Veterinary Medicine, Cairo University, Giza, 12211 Egypt; 4 Department of Pharmacology and Toxicology, Faculty of Pharmacy, Port Said University, Port Said, Egypt; 5 Department of Pharmacology and Toxicology, Faculty of Pharmacy, Al-Azhar University, El-Nasr Road, P.O. 11751, Cairo, Egypt; 6 Department of Pharmacology and Toxicology, Faculty of Pharmacy, Sinai University – Kantara Branch, Ismailia, 41636 Egypt

**Keywords:** Quillaja saponaria, Methotrexate, Pathological kidney, Oxidative stress, Apoptosis, TNF-α/Nrf-2/Keap-1

## Abstract

**Background:**

Methotrexate (MTX) is an antineoplastic/immunosuppressive drug, whose clinical use is impeded owing to its serious adverse effects; one of which is acute kidney injury (AKI). Most of MTX complications emerged from the provoked pro-oxidant-, pro-inflammatory- and pro-apoptotic effects. *Quillaja saponaria* bark saponin (QBS) is a bioactive triterpene that has been traditionally used as an antitussive, anti-inflammatory supplement, and to boost the immune system due to its potent antioxidant- and anti-inflammatory activities. However, the protective/therapeutic potential of QBS against AKI has not been previously evaluated. This study aimed to assess the modulatory effect of QBS on MTX-induced reno-toxicity.

**Methods:**

Thirty-two male rats were divided into 4-groups. Control rats received oral saline (group-I). In group-II, rats administered QBS orally for 10-days. In group-III, rats were injected with single i.p. MTX (20 mg/kg) on day-5. Rats in group-IV received QBS and MTX. Serum BUN/creatinine levels were measured, as kidney-damage-indicating biomarkers. Renal malondialdehyde (MDA), reduced-glutathione (GSH) and nitric-oxide (NO_x_) were determined, as oxidative-stress indices. Renal expression of TNF-α protein and Nrf-2/Keap-1 mRNAs were evaluated as regulators of inflammation. Renal Bcl-2/cleaved caspase-3 immunoreactivities were evaluated as apoptosis indicators.

**Results:**

Exaggerated kidney injury upon MTX treatment was evidenced histologically and biochemically. QBS attenuated MTX-mediated renal degeneration, oxidant-burden enhancement, excessive inflammation, and proapoptotic induction. Histopathological analysis further confirmed the reno-protective microenvironment rendered by QBS.

**Conclusions:**

In conclusion, our results suggest the prophylactic and/or therapeutic effects of QBS in treating MTX-induced AKI. Such reno-protection is most-likely mediated via Nrf-2 induction that interferes with oxidant load, inflammatory pathways, and proapoptotic signaling.

**Graphical Abstract:**

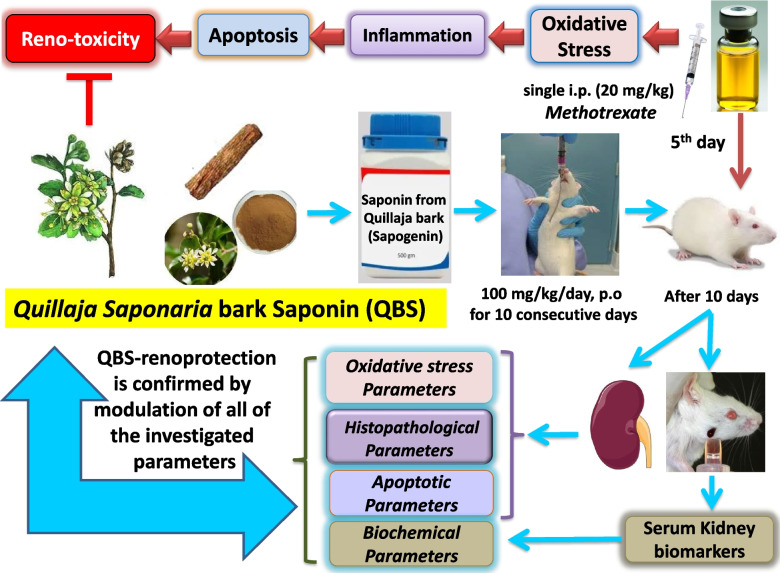

## Introduction

Adverse medications reactions are of the most commonly encountered problems of multiple vital drugs, whose therapeutic applications become significantly limited by such adverse effects [1]. These toxicities mandate prevention/treatment, or alteration of the dose [2]. The chemotherapeutic- and immunosuppressant agent, methotrexate (MTX), is a vivid example of those medications [3]. MTX is a folic acid antagonist with DNA synthesis inhibition [4]. The clinical usage of MTX is limited by its acute vital organ toxicity, which affects the liver, gastrointestinal tract, and bone marrow [3]. With regard to the kidney, acute kidney injury (AKI) is a serious complication in patients on high-dose MTX therapy [5]. Because AKI can delay or terminate therapy with MTX, maximizing its clinical benefits, controlling- and attenuating its associated adverse effects is urgently needed. There are multiple mechanisms of MTX-mediated kidney toxicity, including allergic interstitial nephritis, direct pharmacological toxicity, and precipitation of MTX in renal tubules, which plugs the tubular lumens [6]. MTX-induced nephrotoxicity is commonly induced as a consequence of oxidative stress, suppression of DNA production, inflammatory infiltration, and apoptosis [7]. Under homeostatic circumstances, cells keep the redox microenvironment at a higher reducing power. The change in the redox environment toward a more oxidized potential is referred to as oxidative stress (OS) [8], an important player in the pathophysiology of both, acute- and chronic kidney disease. In AKI, accumulation of toxic end products of nitrogen metabolism and creatinine, and decreased urine output, with rapid loss of kidney function is seen [9]. It was previously reported that MTX induces OS via the over production of reactive oxygen species (ROS), through increased malondialdehyde (MDA), decreased catalase/superoxide dismutase activity, and depletion of reduced glutathione (GSH) in the blood and kidneys [10]. Here comes the role of the key non-enzymatic antioxidant bioactive peptide, GSH, that binds the oxidant substances,, originating substantial antioxidative effects, and preventing renal tubular cell apoptosis [11].

One of the highly reactive ROS is nitric oxide (NO_x_), but with dual activity. In fact, NO_x_ is involved in many physiological and pathophysiological pathways and might either boost or lessen OS-induced cell injury. Beside serving a beneficial role as a messenger and a host defense molecule [12], NO_x_ has been considered as an essential pro-oxidant, since excessive NO_x_ production can be cytotoxic, due to its rection with reactive oxygen/nitrogen species, leading to tyrosine nitration, formation of peroxynitrite anion, and hydroxyl radical [13].

The cells of the immune system are recruited and brought to the site of injury during inflammation, with increasing oxygen consumption, and accumulating of ROS, with oxidative inflammatory consequences [14]. In addition to OS, abnormal generation of inflammatory mediators and neutrophils infiltration contribute to MTX-induced renal damage [15]. As a final response to OS and inflammatory cytokines release, apoptotic cell death is erupted [16]. For the cell to adapt to the oxidative inflammation, the bZIP transcription factor, nuclear factor erythroid 2-related factor-2 (Nrf-2) is activated to contribute to the anti-inflammatory process by transactivation of gene expression of heme oxygenase-1 (HO-1) [17], that has antioxidant outcomes via scavenging peroxy radicals and inhibition of lipid peroxidation [18]. Further, HO-1 negatively regulates several inflammatory mediators, including tumor necrosis factor-alpha (TNF-α) [19]; one of the pro-inflammatory cytokines that are provoked after MTX treatment, leading to neutrophil infiltration and strikes apoptotic cell death [20]. For homeostasis, the negative regulation of Nrf-2 is undertaken by Keap-1, that sequesters Nrf-2 in the cytoplasm, preventing its translocation, leading to low expression of ARE-driven genes [8].

*Quillaja Saponaria Molina* bark saponin (family *Rosaceae*), an evergreen tree native to China and South America, has been used in traditional medicine; orally to relieve cough, and topically to relieve scalp itchiness/dandruff, with additional anti-inflammatory, and immunostimulant activity [21]. The powder of the inner bark of *Q. saponaria Molina* tree, is the primary source of quillaic acid-derived triterpenoids that have been studied for their biological properties including antiviral, antifungal, antibacterial, antiparasitic, and antitumor activities. The *quillaja* bark saponin (QBS) glycoside comprises a hydrophilic sugar and hydrophobic quillaic acid aglycone backbone (sapogenin) [22]. Due to their amphiphilic nature, saponins have the ability to mitigate the effects of the inflammatory mediators [23]. In particular, quillaic acid exhibited strong topical *in-vitro* anti-inflammatory activity as shown in several mouse models [22]. We have recently shown the hepatoprotective effects of QBS in rats [3]. Nevertheless, to our knowledge, no studies have investigated the therapeutic role of QBS in AKI, particularly which is induced by MTX. Importantly, MTX-induced nephrotoxicity could be life-threatening; by delaying elimination of MTX, with further emerging of sustained, elevated circulating MTX level that may provoke other MTX-related toxicities. Due to the complexity of AKI, with contribution of severe endogenous and exogenous morbidity factors, most of the prophylactic measures is solicited to avoid the deterioration of the condition. Toward this end, the present work aimed to explorethe potential prophylactic- and possibly alleviative effects of the saponins derived from QBS against MTX-induced AKI in rat model. We also tried to illustrate some of the potential molecular pathways underlying these effects. In this regard, the biochemical markers illustrating loss of kidney integrity, as serum urea/creatinine were assessed. Tissue redox biomarkers, GSH/MDA/NO_x_ contents, were investigated. Determination of TNF-α/Nrf-2/Keap-1 levels were carried out, to explore the modulation of the OS/inflammation in respective to the designated treatments. Further, assessment of the levels of renal cleaved caspase-3/Bcl-2 immunoreactivities was carried out to determine the extent of apoptosis. Moreover, histopathological studies were performed to inspect the lesions in the adopted kidney tissues.

## Materials and methods

### Ethics statement

The procedures conducted in this study received approval from the '*Research Ethics Committee of the Experimental Animals Use and Care', Faculty of Pharmacy, Beni-Suef University (BSU-IACUC, Egypt); Approval number 022–352* (with additional approval obtained from *Vet, Cairo University, IACUC, Egypt; Vet CU 03162023635*). The guidelines of the 'Animal House Rules follow the conventional guidelines of *National Institutes of Health (NIH).*

### Animals

We got adult male *Wistar* rats weighing 150–180 g were obtained from the *National Research Center, Cairo, Egypt*. Animals were kept in plastic cages and maintained in an animal care facility under standard conditions of controlled temperature (25 ± 1 °C), humidity (50% ± 10%), and 12-h light/dark cycles. They were allowed for free access to standard food and water ad libitum*,* and were left for one week to acclimatize before any experimental procedures.

### Drugs

#### Methotrexate (MTX)

MTX was brought from Baxter Company (Cairo, Egypt) and was given to the rats by single i.p. injection (in a dose of 20 mg/kg at the 5th day).

#### Saponin from Quillaja saponaria Molina

*Quillaja* bark saponin (QBS) powder (S4521) was obtained from Sigma-Aldrich (St. Louis, MO), dissolved in normal saline and given orally to the animals (in a dose of 100 mg/kg/day, for 10 days) [3]. The major aglycone (sapogenin) that is mostly found in QBS is the triterpenoid; quillaic acid (pentacyclic triterpene). It is a hydroxy monocarboxylic acid and an aldehyde consisting mainly of 30-carbon atoms of the Δ^12^-oleanane type (C_30_H_46_O_5_), that was characterized by NMR spectroscopy, mass spectrometry and chemical methods) [24]. The methods for isolation, quantification and quality control of the purified saponin are reported in *Sigma Product information Data Sheet* (S4521).

### Chemicals and kits

N-(1-naphthyl) ethylenediaminedihydrochloride (NEDD), Ellman’s reagent, MDA, reduced GSH and thiobarbituric acid, were obtained from *Sigma-Aldrich* (St. Louis, MO). The 3,3’-diaminobenzidine tetrahydrochloride (DAB) was acquired from *Vector Laboratories Inc*, located in Burlingame, California, USA. Rabbit polyclonal; Bcl-2 antibody (ab194583) and cleaved caspase-3 antibody (NB100-56113) were purchased from *Abcam Biochemicals* and *Novus Biologicals*, respectively. GF-1 Total RNA extraction Kit was obtained from *Vivantis Technologies* (Sdn Bhd, Malaysia). cDNA synthesis Kit was obtained from *Bio-Rad*, CA, USA. All other chemicals used were of the highest standard analytical grade.

### Experimental design

A total of thirty-two rats were categorized into four groups (*N* = *8* each). *Group I* (Control group): represents rats that orally got 0.9% normal saline (vehicle of saponin; 0.2 ml/day, for ten consecutive days), in addition to receiving a single i.p. injection of 0.9% saline (vehicle of MTX) on day-five only. *Group II* (saponin alone-treated group, QBS): includes rats that received oral QBS (100 mg/kg/day) dissolved in normal saline (for ten consecutive days), in addition to a single i.p. injection of 0.9% saline on day-five only. *Group III* (MTX): contains the rats that received oral 0.9% normal saline (for ten days), along with a single i.p. injection of MTX (20 mg/kg) on the 5^th^ day of the trial. *Group IV* (MTX + QBS): comprises the rats that orally administered the saponin (100 mg/kg/day, p.o, for ten days) and a single i.p. dose of MTX (20 mg/kg) at the 5^th^ day of the experiment; 2 h prior to saponin administration. Twenty-four h after the last saponin dose, animals were anesthetized with i.p. thiopental sodium (75 mg/kg) and the blood samples were obtained using heparinized microcapillary tubes from retroorbital plexus, and processed for biomarkers assessments, as previously described [3]. After collection of blood, rats were sacrificed by cervical dislocation and the kidneys were immediately removed and washed three times using ice-cooled normal saline. The right kidneys were fixed in 10% phosphate buffered formalin for histological assessments and immunohistochemical examinations of Bcl-2 and cleaved caspase-3 [25]. The left kidney in each group was divided into two parts; one part was homogenized (1/10 w/v) in ice-cold Tris–HCl buffer (0.1 M, pH 7.4) for the preparation of 10% tissue homogenate and kept in − 20 °C for biochemical assays (determination of oxidative stress and inflammatory markers) [26]. The second part was snap-frozen in liquid nitrogen and kept in − 80 °C for qRT-PCR studies (3 animals/group) for determination of Nrf-2and Keap-1 mRNA expression levels [27].

### Biochemical assays

#### Kidney function analysis

Serum non-protein-nitrogenous substances; BUN (IFU/UREFSR01/00) and creatinine (IFU/CREFSR03/01), were used as biomarkers for kidney function and were estimated spectrophotometrically (UV-1700 Spectrophotometer, Shimadzu, Japan) using commercial kits (*Meril life diagnostic kit*, Gujarat, India) according to manufacturer's instructions [28].

#### Renal oxidative stress assessment

Kidney homogenate (10%) was obtained by adding 1 g of the renal tissue with 9 volumes of ice-cooled phosphate buffered saline (PBS) using IKA homogenizer (*Model T 25 ULTRA-TURRAX*, Staufen, Germany). The resultant homogenates were centrifuged (at 3000 × g), and the attained supernatants were processed using standard methods for determination of renal; GSH, as an index for renal antioxidant activity [29], MDA, as an index of the extent of lipid peroxidation in the kidneys [30], and NO content, as an indicator for oxidant free radicals in renal tissues [31].

### Histopathological and immunohistochemical investigations

#### Histological analysis

Normal saline was used to wash the tissue samples of the kidneys that were fixed in 10% phosphate buffered formalin for 72 h in tightly-sealed containers. Samples were routinely processed in serial grades of ethanol, cleared in Xylene, and then impregnated into paraplast tissue embedding media (*Leica Biosystems*). Paraplast-embedded tissue blocks were cut at 4 μm thickness using rotatory microtome then placed on glass slides and stained with hematoxylin and eosin (H&E) [32]. Tissue sections were investigated under light microscope for demonstration of common indices of kidney histology in different samples, and representative images were shown.

#### Evaluation of apoptosis by immunohistochemical determination of Bcl-2 & cleaved caspase-3

Preparation for immunohistochemical staining, including sections deparaffinization, and antigens retrieving was conducted. After blocking non-specific protein binding, sections were washed by PBS and incubated overnight (at 4 °C) with the primary rabbit antibody against rat; cleaved caspase-3 (NB100-56113) from *Novus Biologicals*, (dilution 1:1000), or Bcl-2 (ab194583) from *Abcam Co* (Cambridge, MA, USA), (dilution 1:100). Then, sections were washed by PBS, incubated with secondary antibody; HRP Envision kit (DAKO), for 20 min, washed by PBS, and positive immunoreactivities were developed by DAB visualization for 10 min and counter-staining with hematoxylin. Quantitative analysis was performed according to El-Nabarawy et. al. (2020) for determination of area percentage of immunohistochemical expression levels of indicated proteins, as estimated from six representative randomly selected fields in the tissue section using Leica application software (*Leica Microsystems GmbH*, Wetzlar, Germany) [33]. Statistical analysis of renal immuno-expressions in different groups was carried out using chi-squared "χ^2^" test. Representative microscopic images (× 400) were shown in the study.

### Evaluation of inflammation

#### Determination of renal mRNA levels of Nrf-2and Keap-1, using Real time PCR

Total RNA was isolated from renal tissues using *GF-1 Total RNA Extraction Kit* (*GF-TR-050, Vivantis Technologies* Sdn Bhd, Malaysia), according to the manufacturer’s instruction. The isolated RNA was treated with a DNase I (*RNase-free kit*; *Fermentas*, MD, USA). After complementary DNA (cDNA) synthesis (*Script™ cDNA synthesis kit*; *Bio-Rad*, CA, USA), the quantitative real time-polymerase chain reaction (qRT-PCR)**,** was conducted as previously described [3]. After PCR amplification, the ΔCt was calculated by subtraction of the β-actin *Ct* from each sample *Ct*., and relative levels of gene expression were determined. The β-actin is used as a reference gene. Table 1 shows the sequences of Nrf-2 and Keap-1 primers.

**Table 1:** The sequences of the oligonucleotide primers used for RT-PCR

Primer	Nucleotide sequence
**Nrf-2**	F: 5′- GCTATTTTCCATTCCCGAGTTAC-3′
	R: 5′-ATTGCTGTCCATCTCTGTCAG-3′
**Keap-1**	F: 5′-GGACGGCAACACTGATTC-3′
	R: 5′-TCGTCTCGATCTGGCTCATA-3′
**β-actin**	F: 5′- CCACCATGTACCCAGGCATT-3′
	R: 5′- ACGCAGCTCAGTAACAGTCC-3′

#### Estimation of renal TNF-α

Along with the modulations of Nrf-2/Keap-1 mRNA levels, the protein level of renal TNF-α was used as a marker for inflammation in the current study. The level of TNF-α in the homogenates of the kidneys tissues were quantitated using ELISA assay (MBS175904), according to the manufacturer’s instructions (*My BioSource*, St. Louis, MO, USA) [34].

### Statistical analysis

Values were reported in the form of the means ± SEM. The various treatments were compared by the *One-way Analysis Of Variance* (ANOVA) test followed by *Tukey–Kramer* comparisons test applied across the four groups or *Chi-Squared* "χ^2^" test wherever indicated. The results were deemed to be significantly different at *p* < 0.05. Data analysis was accomplished using the computer software *GraphPad prism*, SanDiego, USA.

## Results

### Treatment with QBS ameliorated MTX-induced kidney damage and improved renal tissue integrity

**Fig. 1:**
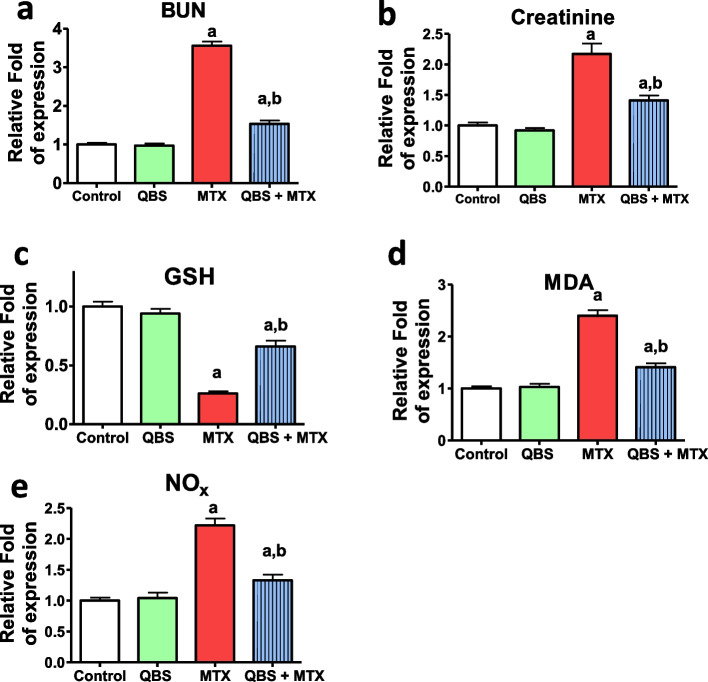
The modulatory effects of QBS pretreatment on the serum levels of the indicated biomarkers in MTX-treated rats. The illustrated data represents the relative fold of expression ± SEM of the levels of non-protein-nitrogenous components [BUN (**a**) & creatinine (**b**)], and renal oxidative stress [GSH (**c**), MDA (**d**) & NOx (**e**)] (*N* = 8). Statistical analysis was carried out using ANOVA followed by Tukey’s multiple comparisons test; ^a^*P* < 0.05, vs. normal control animals, ^b^*P* < 0.05, vs. MTX-treated animals

The determination of the serum levels of urea, and creatinine are routine investigations that used these markers for the evaluation of the state of kidney health/integrity and/or nephrotoxicity (Table 2; Fig. 1a, b). Administration of QBS to rats did not change the levels of the indicated biomarkers compared to normal rats (control group). However, the serum BUN and creatinine in group III were remarkably higher (2.5-fold & 1.2-fold, respectively) in comparison with the corresponding levels in the control group, indicating MTX-induced nephrotoxicity. Interestingly, rats pretreated by QBS exhibited a prominent improvement in renal functions as shown by significant attenuation of MTX-induced elevations in serum levels of BUN (by ~ 56%), and creatinine (by ~ 35%), in comparison with MTX-treated rats. These findings denote the reno-protective effects of QBS against MTX-induced nephrotoxicity.

**Table 2:** The effects of MTX with or without QBS on the biomarkers of kidney functions

Group	Control	QBS	MTX	QBS + MTX
Serum biomarker (mg/dl)				
BUN	34.40 ± 1.76	33.37 ± 2.09	122.6 ± 4.03^a^	53.00 ± 2.69^ab^
Creatinine	0.581 ± 0.026	0.533 ± 0.024	1.262 ± 0.097^a^	0.818 ± 0.049^ab^

The illustrated data represents the means ± SEM of the levels of the indicated biomarkers in the serum of the rats from the indicated groups (*N* = 8). Statistical analysis was carried out using one-way analysis of variance (ANOVA) followed by Tukey’s multiple comparisons test; ^a^*P* < 0.05, vs. normal control animals, ^b^*P* < 0.05, vs. MTX-treated animals

### Treatment with QBS attenuated renal oxidative stress, lipid peroxidation

MTX upregulates ROS that is typically observed in the pathogenesis of AKI, with perturbation of kidney functionality [35]. ROS have a reactive nature with nitric oxide (NO), inducing cell injury. By scavenging ROS and binding toxic electrophilic radicals, GSH can maintain normal cell integrity [36]. However, enhanced ROS production usually occurs in coincide with MTX treatment, which overwhelm the extent of the endogenous antioxidant capacity [37]. In such cases, the successive oxygen free radicals’ chain can give rise to tissues lipid peroxidation with the accumulation of the final product; malondialdehyde, MDA [38]. The current MTX effects on the levels of renal GSH, MDA, and NO_x_ with- and without QBS treatment are shown in (Table 3; Fig. 1c-e). The data showed that the levels of renal GSH, MDA and NO_x_ contents in QBS group was comparable to the untreated group of rats. Group III, however, exhibited substantial decrease (~ 74%) renal GSH content, and significant increase in renal MDA and NO_x_ contents (by about 1.4-fold & 1.2-fold, respectively), in comparison with the normal rats in the control group. Such effects are regarded as a key biomarker for ROS, and are linked to nephrotoxic events of MTX. Pretreatment with QBS showed a potential increase in the hepatic GSH level (by ~ 60%) along with a remarkable decline in both MDA and NO_x_ contents (by ~ 43% & 40% respectively) upon comparison with MTX-treated rats. It is suggested that the reduction of the pro-oxidant load conferred by QBS-mediated improvement of the redox profile is responsible for the renoprotective effects of QBS against MTX-induced kidney damage.

**Table 3:** The effects of MTX with or without QBS on the oxidative stress & inflammatory biomarkers

Group	Control	QBS	MTX	QBS + MTX
Hepatic biomarker content				
GSH (mg/g wet tissue)	0.937 ± 0.041	0.879 ± 0.041	0.243 ± 0.009^a^	0.618 ± 0.048^ab^
MDA (nmol/g wet tissue)	68.10 ± 2.62	70.66 ± 3.94	163.50 ± 7.64^a^	95.79 ± 4.74^ab^
NOx (µmol/g wet tissue)	306.8 ± 15.61	318.3 ± 27.71	680.9 ± 34.11^a^	407.1 ± 26.14^ab^
TNF-α (ng/g wet tissue)	14.68 ± 0.42	8.58 ± 0.46	62.78 ± 2.34^a^	41.30 ± 2.99^ab^

The illustrated data represents the means ± SEM of the levels of the indicated oxidative stress- & inflammatory biomarkers in the renal tissues of the rats in the indicated groups (*N* = 8). Statistical analysis was carried out using one-way analysis of variance (ANOVA) followed by Tukey’s multiple comparisons test; ^a^*P* < 0.05, vs. normal control animals, ^b^*P* < 0.05, vs. MTX-treated animals

### QBS mitigated MTX-induced histopathological degeneration and protect against deteriorating the kidney tissues

**Fig. 2:**
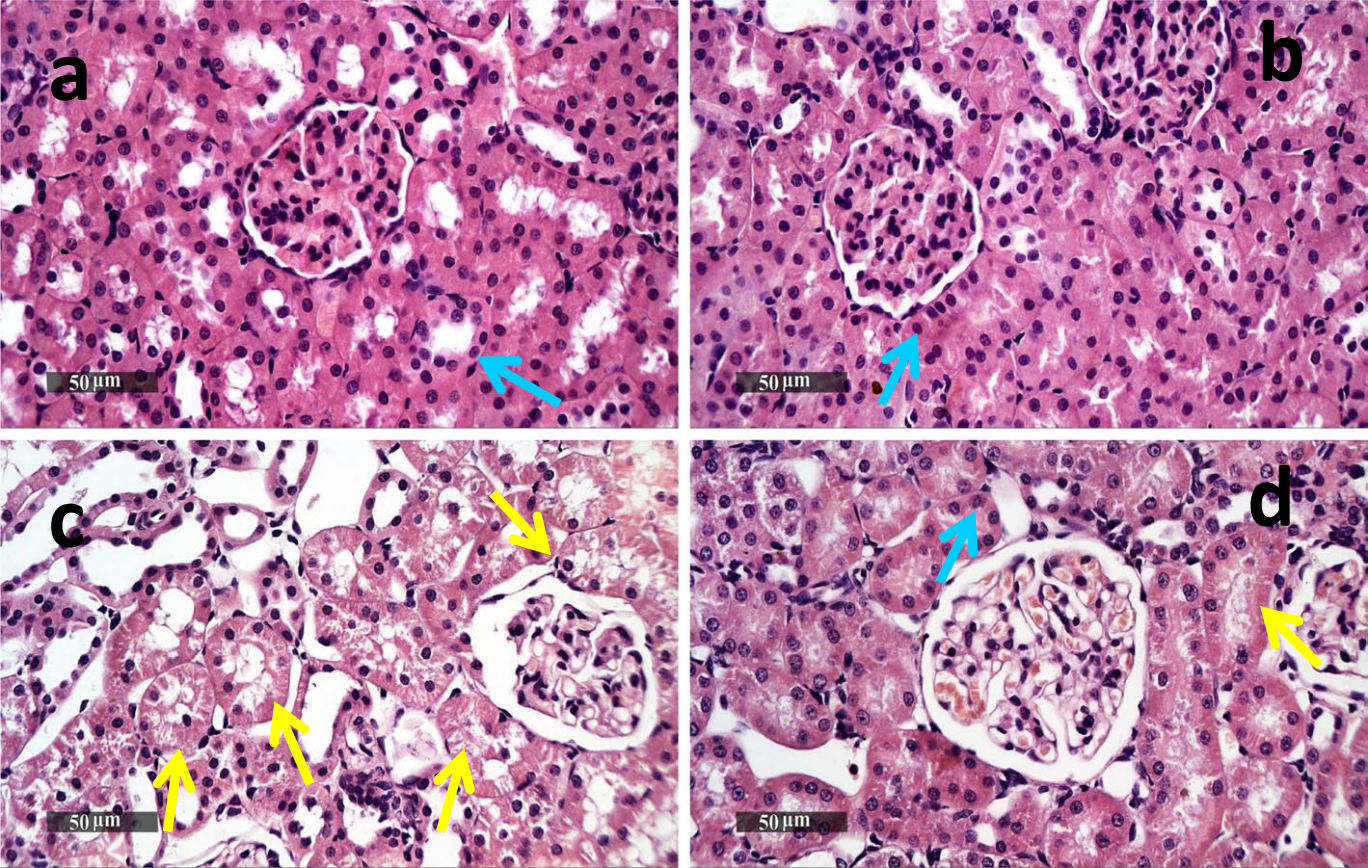
Reno-protective effects of QBS against MTX as shown by microscopic examination of H&E stained-kidney sections with representative microphotographs. **a** & **b** kidney sections from control and QBS groups, respectively, showing normal architecture of renal parenchyma with apparent intact renal; vasculature, corpuscles & tubular epithelia (Blue arrows). Kidney section in (**c**) represents MTX group with marked degenerative changes of renal architecture, shrank glomerular tuft, vascular congestion, scattered inflammatory cells & multiple necrotic tubular epithelia (Yellow arrows). Kidney section in (**d**) represents (MTX + QBS) group; improved renal architecture with only mild vascular congestion & very few necrotic tubular epithelia (Yellow arrows), with almost intact renal tubules. The number of samples examined in each group is 5 (*N* = 5), and representative images are shown (original magnification; 400X)

Effects of MTX administration with- and without QBS treatment on H&E-stained kidney sections are shown in Fig. 2. The microscopic examinations of renal tissues dissected from rats treated with the vehicle only (Fig. 2a) and those treated with QBS only (Fig. 2b) demonstrated normal histological architecture of renal parenchyma with apparent intact; vasculature, renal corpuscles, and renal tubular epithelial cells (Blue arrows). On the contrary, the kidney tissues dissected from MTX-treated rats demonstrated various pathological alterations (Fig. 2c). Such histological changes in renal architecture include; vascular congestion, focal inflammatory cells infiltration, marked degeneration of renal tubules, with spread of necrosis of tubular epithelia (Yellow arrows). The necrotic cells appeared either as homogeneous eosinophilic structure-less masses without any nuclear structure, or with nuclear pyknosis. Also, the renal glomeruli showed shrinkage of the glomerular tuft with dilatation of Bowman's space. Nevertheless, pretreating the rats with QBS remarkably diminished the severity of MTX-induced pathological changes; since the kidney sections obtained from the combination group (QBS/MTX) exhibit improvement in renal architecture with only mild vascular congestion, and very few necrotic tubular epithelia (Yellow arrows), with almost intact renal tubules (Fig. 2d). Taken together, these findings indicate that QBS treatment effectively counteracted MTX-induced kidney damage.

### Effects of QBS treatment on inflammation and Nrf-2/Keap-1 pathway in the kidneys of MTX-treated rats

**Fig. 3:**
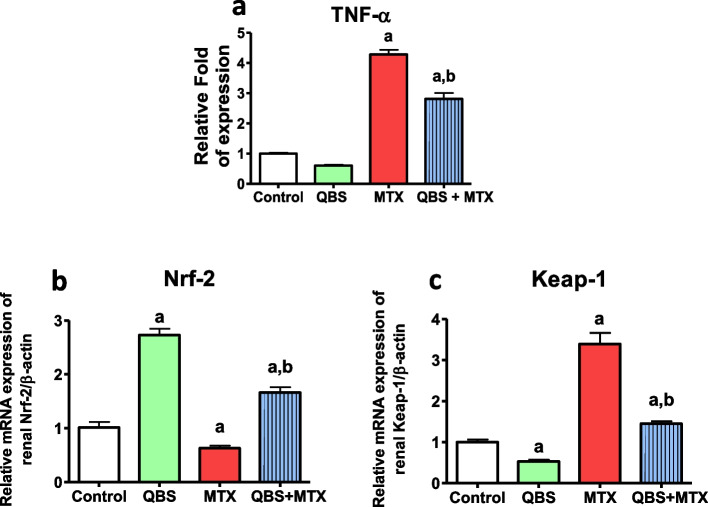
The outcomes of MTX and/or QBS treatments on the renal; protein level of TNF-α, and the mRNA levels of Nrf-2 & Keap-1 in the rats from the indicated groups. The illustrated data represents; **a** the relative fold of expression ± SEM of the levels of renal TNF-α protein (*N* = 8), **b** & **c** renal expression of the indicated mRNAs expressed as means ± SEM of their levels in the indicated groups of rats (*N* = 3). Statistical analysis was carried out using ANOVA followed by Tukey’s multiple comparisons test; ^a^*P* < 0.05, vs. normal control animals, ^b^*P* < 0.05, vs. MTX-treated animals

The deviation in the redox state after MTX treatment characterizes the emergence of renal OS with subsequent induction of inflammation [39]. MTX-induced nephrotoxicity is associated with renal inflammation, with subsequent activation of TNF-α/IL-1β/NF-κB signaling [40]. TNF-α is one of the proinflammatory cytokines that activate NF-κB transcriptional activity of certain proinflammatory genes. Hence, we next examine the expression of TNF-α in the isolated kidney tissues. Our data showed that there was a slight decrease in the basal TNF-α expression in group II, when compared with the untreated group (Fig. 3a). While the levels of TNF-α was markedly higher in MTX group, such elevation was notably abrogated (by ~ 35%) when MTX administration was preceded by QBS treatment. This might be owed to the microenvironment of anti-inflammatory/antioxidant state rendered by QBS, at both normal- as well as proinflammatory conditions. To further confirm the anti-inflammatory outcome of QBS, we further examined the involvement of Nrf-2, which usually contributes to such pathological states [19], to counteract the inflammatory- and apoptotic conditions [41]. Figure 3b, c shows the expression of mRNA levels of Nrf-2 and its negative regulator, Keap-1, a transcription factor that mediates adaptation to OS. Comparing with their basal mRNAs’ levels in untreated control group, QBS treatment exhibited a significant induction of Nrf-2 expression (by about 1.7-fold), with a concurrent 50% suppression of Keap-1 (group II). As for group III, reverse transcriptional effects can be seen (~ 40% decrease in Nrf-2, with ~ 2.4-fold increase in Keap-1), upon comparing to the control group. Then again, QBS pre-treatment mitigated such MTX effects and recorded about onefold increase in Nrf-2 mRNA along with about twofold decrease in Keap-1 mRNA, in the combine group (QBS + MTX), upon comparing with their corresponding levels in MTX group.

### QBS inhibits MTX-induced apoptosis in renal tissues

**Fig. 4:**
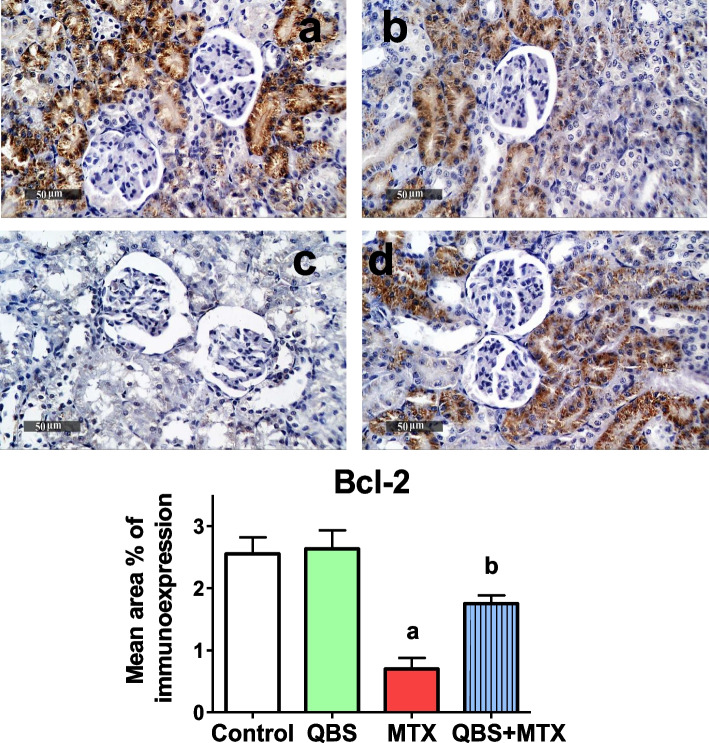
The outcomes of MTX and/or QBS treatments on Bcl-2 immunoreactivity in the kidney sections of the treated rats from the indicated groups. Upper panel: Representative immunostaining images showing mean immunoexpression levels of Bcl-2 of kidney tissue sections from 6 rats in each of control (**a**), QBS (**b**), MTX (**c**) & QBS + MTX (**d**) group. Lower panel: Histograms showing the quantitative analysis of the mean area percentage of Bcl-2 immunohistochemical expression in the examined groups. Each bar represents mean ± SEM of 6 animals in each group (*N* = 6). Statistical analysis was carried out using chi-squared "χ^2^" test; ^a^*P* < 0.05, vs. normal control animals, ^b^*P* < 0.05, vs. MTX-treated animals

**Fig. 5:**
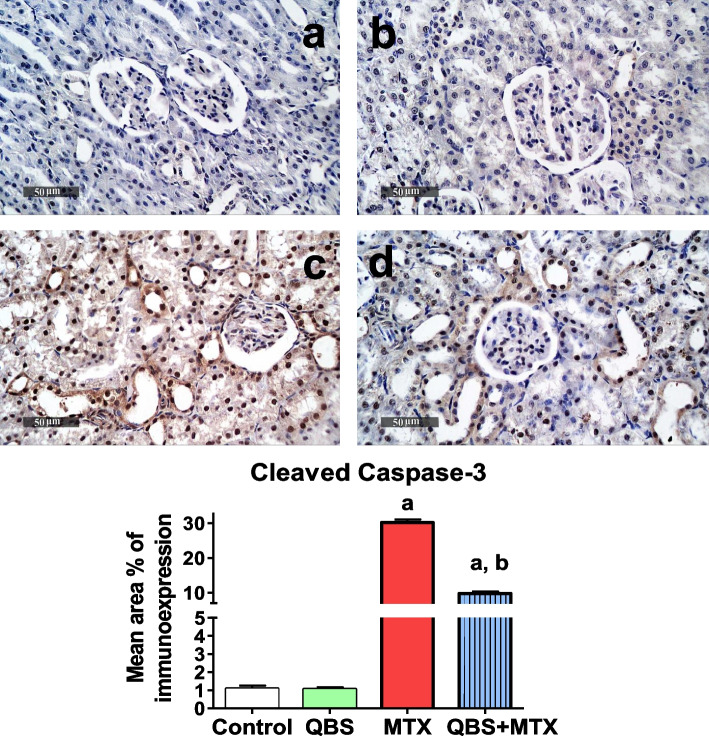
The outcomes of MTX and/or QBS treatments on cleaved caspase-3 immunoreactivity in the kidney sections of the treated rats from the indicated groups. Upper panel: Representative immunostaining images showing mean immunoexpression levels of cleaved caspase-3 of kidney tissue sections from 6 rats in each of control (**a**), QBS (**b**), MTX (**c**) & QBS + MTX (**d**) group. Lower panel: Histograms showing the quantitative analysis of the mean area percentage of cleaved caspase-3 immunohistochemical expression in the examined groups. Each bar represents mean ± SEM of 6 animals in each group (*N* = 6). Statistical analysis was carried out using chi-squared "χ^2^" test; ^a^*P* < 0.05, vs. normal control animals, ^b^*P* < 0.05, vs. MTX-treated animals

The inflammatory cascade is usually engaged with apoptotic cell death. In line, MTX intoxication is usually accompanied with excessive renal apoptosis [1], that might be a sequel to the evoked renal OS and related inflammation [3]. In this work, Bcl-2 and cleaved caspase-3 immunoreactivity were estimated as antiapoptotic- and proapoptotic markers, respectively, to further validate the above-mentioned protective effects of QBS. Our data showed that there was no difference in the expressions of renal Bcl-2 between QBS and control group, with a widespread Bcl-2 immunoreactivity (average mean area precent, MAP of 2.6%; Fig. 4). Also, there was no difference in the expressions of renal cleaved caspase-3 between QBS and control group (average MAP of 1%; Fig. 5). As expected, MTX group exhibited a decrease in renal Bcl-2 expression level (by ~ 1.8-fold; Fig. 4), and recorded the greatest MAP (30%) of renal cleaved caspase-3 immunoreactivity (~ 29-fold increase; Fig. 5), as compared to their basal levels in the respective control groups. Such data constitute the powerful proapoptotic effect of MTX in the inspected rats' kidneys. Yet, QBS pre-treatment attenuated such MTX-induced proapoptotic events. This were evident in the combine group (QBS + MTX) since QBS pretreatment increased renal Bcl-2 expression (Fig. 4), up to ~ 1.8% of the MAP immunoreactivity (onefold more than in MTX group), with a powerful suppression of renal cleaved caspase-3 (Fig. 5), recording about 10% of the MAP immunoreactivity, representing nearly 2-folds less than its level in the MTX group.

## Discussion

MTX is one of the most common and effective immunosuppressants used for treatment of autoimmune diseases [40]. As a classical folate antagonist, MTX is also used in cancer treatment to induce apoptosis in cancer cells. Unfortunately, MTX has the potential to influence cancer cells and normal cells [7]. Therefore, MTX utilization is associated with serious toxic outcomes on several organs, making its clinical use is evidently confined [42]. Owing to the fact that MTX is predominantly excreted by the kidneys by glomerular filtration and active transport, nephrotoxicity represents a common adverse effect [7]. MTX-induced nephrotoxicity occurs when MTX and its metabolites crystallize and precipitate within the renal tubular lumens, along with direct toxicity on mesangial- or tubular epithelial cells, as a result of MTX-enhanced OS and inflammation [40]. MTX-induced nephropathy continues in spite of the preventive measures such as alkalinization of urine and intravenous hydration [41, 42]. With this in mind, this work was conducted to highlight the role of *Quillaja saponaria* bark saponin (QBS) in mitigating MTX-induced AKI. Even though we have recently illustrated that QBS protects against MTX-induced hepatotoxicity [3], with several experimental trials for the prevention of MTX-induced nephrotoxicity, no study to date, have investigated the renal protection of QBS, particularly against maliciousness consequences of MTX on kidneys. In the present study, kidney deterioration was provoked by using 20 mg/kg of MTX, as previously reported [7, 41]. In our study, nephrotoxicity was confirmed by the disturbed renal function (elevated serum creatinine/urea), increasing pro-oxidant load (elevated MDA/NOx with decreased GSH), stimulation of proinflammatory signaling (increased TNF-α/Keap-1 with suppressed Nrf-2), and with the activation of apoptosis (upregulation of cleaved caspase-3, and downregulation of Bcl-2). Our findings manifested QBS powerful protection against MTX-induced renal damage, with an ample clue of associated inhibition of OS and inflammation.

In nephrotoxicity, ROS are generated with exhaustion of antioxidant enzymes, including superoxide dismutase (SOD), catalase, and glutathione reductase [9]. The upregulated ROS that is typically observed in AKI renders renal oxidative damage, and perturbs kidney functionality, the effects that are observed in coincide with MTX treatment [35]. Although GSH maintains normal renal cell integrity by scavenging ROS and electrophilic radicals [36], excessive ROS production in renal tissues could overwhelm the endogenous antioxidant capacity [37], leading to renal lipid peroxidation [38]. MTX potentiality destructs cells via production of excessive ROS, resulting in attenuation of the efficiency of antioxidant enzymes, and ending with apoptosis [35]. Antioxidants interfere with the early stages of AKI pathogenesis, by direct elimination of ROS or the oxidant source. Because saponins have scavenging activity for excessive radicals [43], it is assumed that the protective effect of QBS is attributed to its antioxidant characteristics, which hinder the emerged tissue damage, as several other saponins [43–45].

It is worth noting that the mechanism of MTX in alleviating rheumatoid arthritis and dermatitis is not entirely verified. Whether MTX reduces systemic inflammation and acquires antioxidant features or confers pro-oxidant inflammation is still a debated issue. MTX was previously shown to inhibit OS via scavenging specific types of free radicals [46]. Conversely, most of the other studies demonstrated MTX-related renal OS, inflammation and tubular apoptosis [1, 41, 42], and our results are in harmony with these studies’ findings. Another debatable finding in our work is the effect of MTX on NO_x_, one of the biological modulators of kidney function. The reactive nature of NO_x_ with ROS indicates several pathways through which, NO_x_ may either enhance or attenuate OS-induced cell injury [46]. Although NO_x_ has several valuable functions including the regulation of renal hemodynamics, mediation of pressure-natriuresis, modulation of tubular sodium reabsorption and renal sympathetic neural activity [12], it was previously reported that NO_x_ levels were significantly increased in renal diseases, and were correlated with the serum creatinine/urea concentration. This is assumed to be due to stimulation of cytokine-induced NO_x_ synthase and platelets-mediated NO generation due to uraemia [13]. In our work, the levels of renal NO_x_ were high in MTX-treated rats, and were correlated with the high levels of renal MDA, and serum urea/creatinine in the same rats. In the co-treatment group, QBS counteracts the aforementioned events as manifested by a substantial rise in kidney GSH, in accompany with powerful decreases in renal MDA/NO_x_ contents and serum creatinine/urea, upon compared to their respective levels in MTX group. In fact, the duality of NO’s beneficial and detrimental effects deserves the contemplation of this molecule with the need for a detailed understanding of its pathophysiology. Because inflammation could generate oxidant load with diminishing cellular antioxidant capacity, it is assumed that inflammation and OS, are closely related and simultaneously occurred processes [39]. It was previously reported that, inflammation-induced upregulation of inducible *nitric oxide synthase* leads to the production of excessive NO, generating highly reactive superoxides by oxidation of oxygen. This excessive NO competes with *SOD* and reacts with superoxide radicals creating peroxynitrite, which has a direct damaging effect on tubular cells [9]. These findings support our data concerning overproduction of NO and inflammation eruption after MTX treatment, the events that were abated upon combining with QBS.

When it comes to our attempts to explore the mechanism of QBS activity, our study explored the possible involvement of Nrf-2, in opposing the induced OS/inflammation. The Nrf-2 contributes to the anti-inflammatory process by regulating gene expression through the ARE, including HO-1, and coordinating the recruitment of inflammatory cells [17]*.* Thereupon, Nrf-2 plays a pivotal role in antagonizing OS, with subsequent potent anti-inflammatory effects [47]. As a defensive pathway, Nrf-2 is activated under mild/moderate OS conditions, where ROS dissociate Nrf-2 from its negative regulator, Keap-1, and translocate into the nucleus to activate the transcription of ARE, to counteract the apoptosis and several inflammatory mediators as NF-κB and TNF-α [19]. In contrast, severe and excessive OS resulted in repression rather than activation of Nrf-2 signaling [48]. Interestingly, impaired Nrf-2 activity was seen in rats with kidney injury, with progression of glomerulosclerosis, tubulointerstitial fibrosis, proteinuria, and renal insufficiency that were accompanied by NF-κB activation [49]. In agreement with that notion, our results demonstrated that MTX significantly reduced renal Nrf-2- and increased Keap-1 (at transcription level), imposing robust proinflammatory and pro-oxidant burden on the kidneys. Treatment with QBS alone readjusted the basal redox state of the renal microenvironment at a higher reduction potential, as a result of enhancing renal Nrf-2 transcription and detracting Keap-1 transcription. This gave rise to a primed anti-oxidant potential, which upon combination with MTX, inclusively attenuated the erupted MTX consequences. In this context, QBS-mediated enhancement of the profile of kidney function/histology could be explained on the basis of restoring the redox balance that renders anti-oxidant state, and repairs the accumulated oxidative damage during stress, exhibiting regeneration responses. This was reflected by our histopathological examinations, since MTX produced renal degenerative lesions, and inflammatory reaction, as previously reported [1]; the effects that were notably lower upon combining with QBS. It is noteworthy that during the inflammation process, the evolution of apoptosis is usually developed as a result to the engagement of the involved cells with their inflammatory surroundings [1]. As a proof, the proinflammatory cytokine, TNF-α, activates NF-κB to translocate to the nucleus, and induce transcription of genes responsible for initiating downstream signaling of programmed cell death [40]. The anti-apoptotic, Bcl-2 is an arbiter of the suppression of apoptosis, by inhibiting the activity of pro-apoptotic Bcl-2-associated-X-protein (Bax), repressing the propagation of intrinsic apoptotic pathway with concurrent modulation of caspases activation. Bcl-2 inhibits procaspase-3 activation by preventing cytochrome c release from the mitochondria. The dysregulation of the balance between pro- and anti-apoptotic Bcl-2 family members was found to be correlated with increased apoptosis in polycystic rat kidneys [50]. Should inflammation and apoptosis be closely related, we investigated the amendment of apoptotic signaling, by investigating the immunoreactivity of renal Bcl-2/cleaved caspase-3 proteins. MTX produced proapoptotic profile through suppressing renal Bcl-2, with consequent caspase-3 activation. Such events were significantly lessened by the prior saponin administration. To this end, the repression of apoptosis could be conspicuously related to the activation of Nrf-2 signaling with the ensuant preclusion of MTX-oxidative hazards. Our results agree with several recent findings: suppression of inflammation/apoptosis with renoprotective effect of glycyrrhizin, a triterpene glycoside saponin, after MTX intoxication [51]; attenuation of MTX-induced OS-mediated kidney damages by dioscin saponin [45]; regression of apoptosis and testicular damage by the new saponin, zygo-albuside-A, against MTX [52]. Nonetheless, given the fact that the anti-rheumatoid and antineoplastic effects of MTX could be attributed to eruption of apoptosis [53], attenuating MTX- induced apoptosis by add-on drugs/herps and its impact on the clinical outcome of MTX could be a paradox. Further long-term studies on rodents’ models with cancer or RA are needed.

## Conclusion

**Fig. 6:**
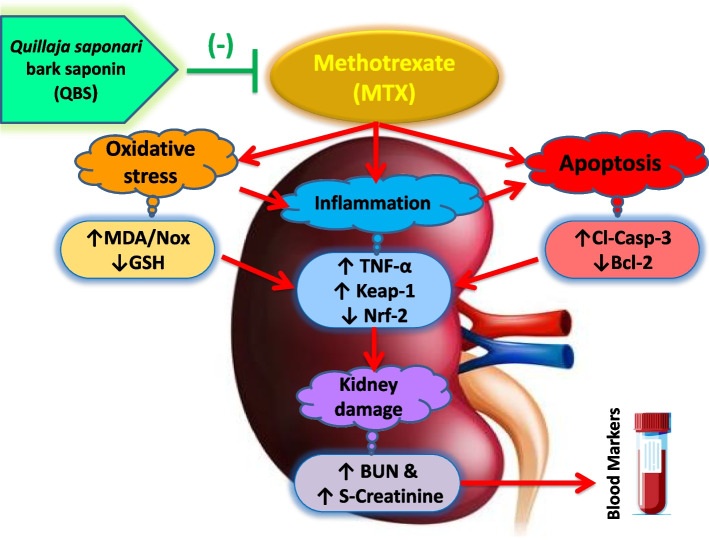
The postulated mechanisms of Quillaja saponaria bark saponin (QBS) in alleviating methotrexate (MTX)-induced renal toxicity in rats. QBS administration mediates reno-protection by adjusting redox state of the renal microenvironment at a higher reduction potential. This renders a powerful conservancy of the renal tissues against MTX, as confirmed by the improved kidney histology & function profile; significant fall in the serum non-protein-nitrogenous components (BUN & creatinine). Such QBS-mediated protection is suggested to be maintained via attenuation of MTX-induced renal; OS (↑GSH/↓MDA/↓NO_x_ → ↓ROS), & inflammation signaling (↓TNF-α/↑Nrf-2/↓Keap-1), with consequent suppression of cell death signaling (↑Bcl-2/↓ cleaved caspase-3)

In summary, the current study provides sufficient evidence that QBS co-administration with MTX brings about significant conservation of the kidneys by attenuating OS, counteracting pro-inflammatory pathways, and suppressing the ensuing activation of pro-apoptotic signaling. Such events confer renal protection that was manifested on biochemical, molecular, and histopathological scales. Being a clinically-used in traditional medicine, this saponin could be proposed as a prospective dietary supplement promoting renal health, after elaborating clinical studies in patients on MTX therapy with suspected kidney hazards. Figure 6 delineates a summary of the proposed QBS-renoprotective effects.

## Data Availability

The datasets generated during and/or analyzed during the current study are available from the corresponding author on reasonable request.

## Abbreviations

AKI: Acute kidney injury
BUN: Blood urea nitrogen
ARE: Antioxidant responsive element
Bcl-2: B-cell lymphoma-2, anti-apoptotic protein
GSH: Reduced-glutathione
H&E: Haematoxylin–eosin
HO-1: Heme oxygenase-1
Keap-1: Kelch ECH associating protein-1
MDA: Malondialdehyde
MTX: Methotrexate
NO_x_: Total nitric-oxide content
Nrf-2: Nuclear factor erythroid 2-related factor-2
OS: Oxidative stress
QBS: *Quillaja saponaria* Bark saponin
RA: Rheumatoid arthritis
ROS: Reactive oxygen species
TNF: Tumor necrosis factor
